# The Effect of Gut Microbiome Composition on Human Immune Responses: An Exploration of Interference by Helminth Infections

**DOI:** 10.3389/fgene.2019.01028

**Published:** 2019-11-06

**Authors:** Ivonne Martin, Maria M. M. Kaisar, Aprilianto E. Wiria, Firdaus Hamid, Yenny Djuardi, Erliyani Sartono, Bruce A. Rosa, Makedonka Mitreva, Taniawati Supali, Jeanine J. Houwing-Duistermaat, Maria Yazdanbakhsh, Linda J. Wammes

**Affiliations:** ^1^Department of Mathematics, Faculty of Information Technology and Science, Parahyangan Catholic University, Bandung, Indonesia; ^2^Department of Biomedical Data Sciences, section Medical Statistics, Leiden University Medical Center, Leiden, Netherlands; ^3^Department of Parasitology, Faculty of Medicine, Universitas Indonesia, Depok, Indonesia; ^4^Department of Microbiology, Faculty of Medicine, Universitas Hasanuddin, Makassar, Indonesia; ^5^Department of Parasitology, Leiden University Medical Center, Leiden, Netherlands; ^6^McDonnell Genome Institute, Washington University in St. Louis, St. Louis, MO, United States; ^7^Department of Medicine, Washington University School of Medicine, St. Louis, MO, United States; ^8^Department of Statistics, University of Leeds, Leeds, United Kingdom; ^9^Department of Biostatistics and Research Support, Julius Center, University Medical Centre Utrecht, Utrecht, Netherlands; ^10^Alan Turing Institute, London, United Kingdom

**Keywords:** helminth, gut microbiome, whole blood cytokine, interleukin-10, *Bacteroidetes*, diversity, randomized controlled trial

## Abstract

**Background:** Soil-transmitted helminths have been shown to have the immune regulatory capacity, which they use to enhance their long term survival within their host. As these parasites reside in the gastrointestinal tract, they might modulate the immune system through altering the gut bacterial composition. Although the relationships between helminth infections or the microbiome with the immune system have been studied separately, their combined interactions are largely unknown. In this study we aim to analyze the relationship between bacterial communities with cytokine response in the presence or absence of helminth infections.

**Results:** For 66 subjects from a randomized placebo-controlled trial, stool and blood samples were available at both baseline and 21 months after starting three-monthly albendazole treatment. The stool samples were used to identify the helminth infection status and fecal microbiota composition, while whole blood samples were cultured to obtain cytokine responses to innate and adaptive stimuli. When subjects were free of helminth infection (helminth-negative), increasing proportions of *Bacteroidetes* was associated with lower levels of IL-10 response to LPS {estimate [95% confidence interval (CI)] −1.96 (−3.05, −0.87)}. This association was significantly diminished when subjects were helminth-infected (helminth positive) (*p*-value for the difference between helminth-negative versus helminth-positive was 0.002). Higher diversity was associated with greater IFN-γ responses to PHA in helminth-negative (0.95 (0.15, 1.75); versus helminth-positive [−0.07 (−0.88, 0.73), *p*-value = 0.056] subjects. Albendazole treatment showed no direct effect in the association between bacterial proportion and cytokine responses, although the *Bacteroidetes*’ effect on IL-10 responses to LPS tended downward in the albendazole-treated group [−1.74 (−4.08, 0.59)] versus placebo [−0.11 (−0.84, 0.62); *p*-value = 0.193].

**Conclusion:** We observed differences in the relationship between gut microbiome composition and immune responses, when comparing individuals infected or uninfected with geohelminths. Although these findings are part of a preliminary exploration, the data support the hypothesis that intestinal helminths may modulate immune responses, in unison with the gut microbiota.

**Trial Registration:** ISRCTN, ISRCTN83830814. Registered 27 February 2008 — Retrospectively registered, http://www.isrctn.com/ISRCTN83830814.

## Introduction

Diseases of modernity, such as allergy, autoinflammatory, and metabolic diseases are increasingly observed in industrialized countries. It has been speculated that this growing rate was caused by changes in lifestyle, diet and environmental factors, such as pollutant exposure or hygiene. Hygiene improvement has dramatically decreased the prevalence of certain infectious agents such as parasitic helminths while these may have protective effects against autoinflammatory diseases (Wammes et al., 2016). Studies analyzing the capacity of helminths to modulate the immune system have been carried out in recent decades. However, it has become clear that this is an interplay with several other factors, such as diet, environment and also other gut inhabitants, such as the microbiota.

Early studies showed that gut microbiota is involved in developmental aspects of the immune system and that disturbance can lead to autoinflammatory disorders (Round and Mazmanian, 2009). Already in 1963, it was reported that the immune system of germ-free mice failed to respond to molecular patterns of pathogenic and beneficial microorganisms, causing morphological tissue defects in the intestinal wall (Abrams et al., 1963). In healthy humans, the role of gut microbiota and immune response was studied more recently. It was found that certain bacteria are beneficial for development and function of the immune system and simultaneously the immune system can influence the composition or function of gut microbiota, all relating to inflammatory disorders [reviewed in Belkaid and Hand (2014)].

The presence of parasitic helminths in the gastrointestinal tract may exert a direct influence on the host’s gut microbiome as they share the same niche. Although in animal models helminths were shown to increase microbial abundance and diversity (Reynolds et al., 2015), the findings in human studies are not consistent. Several studies analyzing the effect of helminth on gut microbiota have indicated a higher diversity of gut microbiota in helminth-positive subjects compared to helminth-negative subjects (Lee et al., 2014; Ramanan et al., 2016). A study in Ecuador showed that this difference in diversity might be related to specific helminth species since they did not find any alterations in *Trichuris trichiura*-infected children (Cooper et al., 2013). This might be influenced by different factors among which are different bacterial profiling techniques or confounders such as ethnicity, anthelminthic treatment, and environmental differences.

As it has been shown that changes in both gut microbiota and helminth infection status might affect the host’s immune response, it is suspected that the presence of helminth might directly or indirectly affect the immune system by altering the gut microbial community (Zaiss et al., 2015). For instance, the transfer of the microbiota of *Heligmosomoides polygyrus bakeri*-infected mice to uninfected mice induced similar protection against allergic airway inflammation as observed with helminth infection (Wilson et al., 2005). In humans, studies on the triangular relation between helminth with the microbiome and immune system are still in infancy.

To our knowledge, the number of longitudinal studies analyzing the association between gut microbiota and immune responses in helminth-endemic areas is still limited. To understand the interaction of the gut microbial community and helminths and their common effect on immune responses, we used data from a household cluster-randomized, double blind, placebo-controlled trial of albendazole treatment in a helminth-endemic area. In this study, it has been shown that deworming reduced helminth prevalence and consequently increased several whole blood cytokine responses (Wammes et al., 2016). Helminth infection and anthelminthic treatment separately did not change the gut microbiota (Martin et al., 2018). However, when subjects remained infected while treated with albendazole, a decrease of *Bacteroidetes*: *Firmicutes* ratio and an increase of *Actinobacteria*: *Firmicutes* ratio were observed, leading to the hypothesis that there is a cross-talk between microbiome composition and immune response which is disrupted by the presence of helminths and that removing helminth by anthelminthic might affect this communication. Our aim was to characterize the association between bacterial relative abundance with the whole blood cytokine responses and the effect of helminth infections and deworming on this interaction.

## Materials and Methods

### Participants

Stool samples from 150 subjects from the immunoSPIN study (Wiria et al., 2010) taking place in Ende subdistrict, Indonesia, were analyzed for the fecal microbiome. From these 150 subjects, 66 subjects were included in this study based on the complete stool data and available cytokine measurements before and 21 months after the first treatment. The microbiome composition for these subjects are representative of these 150 subjects (**Table S1**). Four different helminth species were found namely *Ascaris lumbricoides*, hookworms (*Necator americanus* and *Ancylostoma duodenale*) and *T. trichiura*. Details on sample collection and measuring the infection status using PCR are described elsewhere (Wiria et al., 2010). *T. trichiura* infection was assessed only by microscopy, since at that time there was no real-time PCR data available for this species. For this manuscript, we defined a helminth-infected subject as a participant with a positive real-time PCR (cycle of threshold (Ct) value ≤ 30) and/or positive microscopy for one or more species of helminths, as described previously (Martin et al., 2018). Subjects with a positive real-time PCR with a Ct above 30 were regarded as uninfected.

### Microbiome Composition

The amplification and pyrosequencing of the 16S rRNA gene followed the protocols developed by the Human Microbiome Project (HMP) (A framework for human microbiome research. Nature, 2012) at the McDonnell Genome Institute, Washington University School of Medicine in St. Louis and have been described previously elsewhere (Martin et al., 2018; Rosa et al., 2018). Briefly, the V1–V3 hypervariable region was PCR — amplified and the PCR products were sequenced on the Genome Sequencer Titanium FLX (Roche Diagnostics, Indianapolis, Indiana), generating on average 6,000 reads per sample. Details of the filtering and analytical processing of 16S rRNA data for this cohort has been previously described in Rosa et al. (2018). The assembled contigs count data as a result of RDP classification was organized in a matrix format with taxa in columns and subjects in rows. The entries in the table represent the number of reads for each taxon for each subject. Our work is focused at a phylum level of gut bacteria. Five bacterial phyla have average relative abundances larger than 1%, namely *Actinobacteria*, *Bacteroidetes*, *Firmicutes*, *Proteobacteria* and an unclassified category, which consists of sequences which could not be categorized into a phylum. The remaining bacterial phyla which had lower relative abundance were pooled together into a pooled category. In the analysis, we retained the count for the three most abundant bacterial phylum proportions, namely *Actinobacteria*, *Bacteroidetes*, and *Firmicutes*. The proportion for each phylum was obtained by dividing each sequence count by the total sequence per person at each time point. Along with bacterial proportions, we computed at a phylum level the bacterial diversity within samples (Shannon index) and between samples (Bray-Curtis dissimilarity) using R package vegan (Oksanen et al., 2017). The Shannon index represents not only the presence of taxa but also the abundance of corresponding taxa. The higher diversity index means that there was not a single taxon dominating the community and the total bacterial abundance is spread out over all taxa. The Bray Curtis dissimilarity measures the percentage of similarity between one sample from the other with values range from 0 (completely similar) to 1 (completely dissimilar).

### Whole Blood Cytokine Responses

The method to obtain and assess the cytokines responses were described elsewhere (Wiria et al., 2010). In brief, heparinized blood was diluted 1:4 and cultured in 96-well plates. Plates were incubated for 24 (innate responses) or 72 (adaptive responses) hours, after which supernatants were harvested and stored in freezers. Cytokine levels were measured by Luminex bead technology in samples obtained at before and 21 months after the start of treatment.

The analyses carried in this manuscript are limited to innate responses [interleukin (IL)-10 and tumor necrosis factor-alpha (TNF-α)] to lipopolysaccharide (LPS) from *E. coli* and adaptive responses [interferon-gamma (IFN-γ) and IL-5) to *Ascaris* antigen (AscAg) and general T cell stimulator phytohemagglutinin (PHA)]. The AscAg was a homogenate of adult worm *A. lumbricoides* obtained from infected human (Wammes et al., 2014).

### Statistical Method

All parameters of interest were described as means or frequency (± standard deviation). Prevalence rates were calculated and compared using the Pearson chi-square test, while the Student *t*-test was used to compare continuous variables.

To study the relationship between cytokines and microbiome over the two time points, a linear mixed effect regression model was fitted with helminth status and treatment as covariates. All models have been adjusted with age and sex, however, since both covariates were not significantly associated with the cytokine responses in any model, they are not included in the final analysis. The correlation between observations from the same subjects was modeled by including a subject-specific random effect. The microbiome was included in the model either as a bacterial proportion or by the Shannon diversity index. The cytokine responses were log_10_-transformed [log_10_(concentration + 1)] to obtain normally distributed variables. First, we analyzed the main effect of bacterial proportion and diversity on cytokine responses. Second, to allow for different effect sizes of bacterial proportion or diversity on cytokine responses in helminth-positive versus -negative subjects, an interaction term between bacterial categories and infection was included in the model. The *p*-value for this interaction term indicated the statistical evidence for different effect sizes in helminth-positive or -negative groups. Due to limited sample size, we restricted the analysis into estimating the general effect of helminth infection on the relationship between bacterial proportion and cytokine responses. In the same manner, due to the limitation of sample size, the direct treatment effect cannot be estimated. This means that the estimates for the infection effect on the relationship between bacterial proportions and diversity on cytokine responses were obtained from data of subjects who were infected at any time point, regardless of their randomization arm. However, as treatment removes helminth infection, the analysis of treatment effect on the relationship between bacterial proportion and cytokine responses is viewed as a proxy to understand the role of helminth in this relationship.

For this purpose, a linear mixed effect model was fitted with bacterial proportion or diversity, treatment, and time as covariates. This model was able to characterize three different associations, namely the association between bacterial proportion or diversity on cytokines at pre-treatment, the difference of the association at pre-treatment, and at post-treatment in the placebo group (time effect), as well as the difference of the association at post-treatment between albendazole and placebo group.

For each outcome separately, these models were fitted on subjects who at least had an observation at pre-treatment. The lme4 package in statistical software R was used for model fitting. The significance of the covariate effect was obtained from the likelihood ratio test. Bonferroni correction was used to adjust for multiple testing. We have applied Bonferroni correction for the number of non-correlated cytokine per stimulatory condition resulting in dividing the alpha cut-off level for significance by 2 (LPS responses) and 3(AscAg responses) and 2(PHA responses). The statistical analyses were performed in R (R Core Team, 2017) with mainly lme4 and lmerTest packages (Bates et al., 2015; Kuznetsova et al., 2017). The full record was created using the knitr package in R (Xie, 2017) and is available online at https://github.com/Helminths_GutMicrobes_Cytokine/.

## Results

### The Effect of Bacterial Proportions and Diversity on *In Vitro* Cytokine Responses

Since it is hypothesized that gut bacteria are associated with certain cytokine responses and thereby possibly immune disorders, we set out to explore this relationship by using data from the ImmunoSPIN trial. For 66 subjects, cytokine responses were measured at pre-treatment and 21 months after the start of anthelmintic treatment. At baseline, 40 out of 66 (60.6%) individuals in Ende were infected with one or more helminth species, and hookworm was the most dominant species (31.8%) followed by *A. lumbricoides* (25.7%) and *T. trichiura* (22.7%). The baseline characteristics such as age, gender, BMI and helminth prevalence were similar between the two treatment arms (**Table 1**). Three-monthly albendazole treatment for 21 months reduced the infection prevalence from 65.4% to 19.2% versus a slight increased of helminth infections from 57.5% to 65% in the placebo group (**Table S2**).

**Table 1:** Characteristics of the participants at baseline.

Characteristics		Albendazole		Placebo	
		N	Result	N	**Result**
Gender, female [N (%)]		26	12 (46.1)	40	22 (55.0)
Age [mean (SD)]		26	27.3 (16.1)	40	26.7 (15.7)
Children [< = 18 years old; N (%)]			10 (38.4)		17 (42.5.0)
Adults [>18 years old; N (%)]			16 (61.5)		23 (57.5)
zBMI [mean (SD)]		10	−0.52 (0.98)	17	−0.83 (0.64)
BMI [mean (SD)]		16	23.39 (3.44)	23	23.49 (4.89)
**Parasite infection [N (%)]**					
*A. lumbricoides* *^a^*		26	9 (34.6)	40	8 (20.0)
Hookworm		26	11 (42.3)	40	10 (25.0)
*N. americanus* *^a^*		26	10 (38.5)	40	10 (25.0)
*A. duodenale* *^a^*		26	2 (7.7)	40	2 (5.0)
*T. trichiura* *^b^*		26	5 (19.2)	40	10 (25.0)
Any helminth		26	17 (65.4)	40	23 (57.5)
**Abundance of bacterial phyla [mean % (SD)]**		26		40	
*Actinobacteria*			9.1 (5.8)		9.6 (7.7)
*Bacteroidetes*			8.0 (10.5)		7.1 (12.1)
*Firmicutes*			72.4 (10.4)		71.6 (12.8)
*Proteobacteria*			8.5 (7.3)		8.6 (6.2)
unclassified bacteria^#^			1.4 (0.8)		2.5 (3.1)
pooled*			0.6 (0.8)		0.6 (0.9)
**Diversity Index, median (IQR)**		26		40	
Shannon index			0.85 (0.71, 0.99)		0.84 (0.73, 1.00)
Bray-Curtis			0.19 (0.12, 0.26)		0.19 (0.13, 0.28)
**Cytokine responses (pg/mL, median, IQR)**					
LPS	TNF-α	25	664.00 (294.00,1029.00)	40	550.50 (343.00, 840.00)
	IL-10	25	242.00 (132.00, 400.00)	40	213.50 (142.00, 380.20)
AscAg	IFN-γ	23	28.50 (12.10, 111.80)	37	17.40 (7.74, 60.90)
	IL-5	22	32.55 (9.55, 58.42)	37	18.90 (12.00, 62.00)
	IL-10	23	8.28 (3.22, 22.45)	37	6.61 (2.55, 10.30)
PHA	IFN-γ	23	2,449.00 (354.00, 5,424.00)	37	2299.00 (997.00, 3829.00)
	IL-5	23	490.00 (276.00, 747.50)	37	515.00 (333.00, 870.00)
	IL-10	23	101.00 (54.05, 167.00)	37	83.20 (36.80, 140.00)

^a^Diagnosed by real-time PCR; ^b^diagnosed by microscopy; ^#^unclassified bacteria represents the category of sequences that could not be assigned to a phyla, and the; *pooled category consists of the remaining 13 phyla with average relative abundance less than 1%.

We analyzed proportions of three bacterial phyla (*Actinobacteria*, *Bacteroidetes*, and *Firmicutes*) as these were most abundant in our study population. For the cytokine responses, we selected outcomes representative of different parts of the immune system. We have opted for the pro- and anti- inflammatory (TNF and IL-10 respectively) immune response to LPS to represent the innate responses, and the Th1 and Th2 signature cytokines (IFN-gamma and IL-5 respectively) for the adaptive response to a helminth antigen (AscAg) and T-cell stimulatory PHA. When fitting the linear mixed model, no direct effect was observed of bacterial proportions or Shannon diversity on whole blood cytokine responses (**Table 2**).

**Table 2:** The association between bacterial proportion and diversity on cytokine responses.

		Estimated effect (95% CI)			
		*Actinobacteria*	*Bacteroidetes*	*Firmicutes*	Shannon
**LPS**	**IL-10**	0.20 (−0.58, 0.98)	−0.39 (−0.90, 0.12)	0.24 (−0.23, 0.71)	−0.22 (−0.51, 0.07)
	**TFN-α**	0.55 (−0.35, 1.44)	−0.06 (−0.66, 0.54)	−0.14 (−0.70, 0.41)	0.03 (−0.31, 0.37)
**AscAg**	**IFN-γ**	−1.03 (−2.45, 0.39)	0.15 (−0.80, 1.10)	−0.20 (−1.13, 0.74)	0.14 (−0.44, 0.71)
	**IL-5**	−1.02 (−2.78, 0.74)	0.09 (−1.10, 1.28)	0.39 (−0.74, 1.52)	−0.48 (−1.16, 0.20)
	**IL-10**	−0.04 (−0.56, 0.48)	0.15 (−1.21, 1.51)	−0.72 (−1.63, 0.18)	0.30 (−0.57, 1.16)
**PHA**	**IFN-**γ	−0.57 (−2.12, 0.98)	−0.26 (−1.28, 0.75)	−0.03 (−1.05, 0.99)	0.45 (−0.18, 1.08)
	**IL-5**	−0.04 (−1.55, 1.46)	0.32 (−0.67, 1.32)	−0.85 (−1.82, 0.11)	0.61 (0.02, 1.20)
	**IL-10**	0.46 (0.02, 0.91)	0.33 (−0.80, 1.46)	−0.03 (−0.78, 0.72)	−0.46 (−1.19, 0.27)

### Interference by Helminth Infection in the Effect of Bacterial Proportions and Diversity on *In Vitro* Cytokine Responses

**Figure 1:**
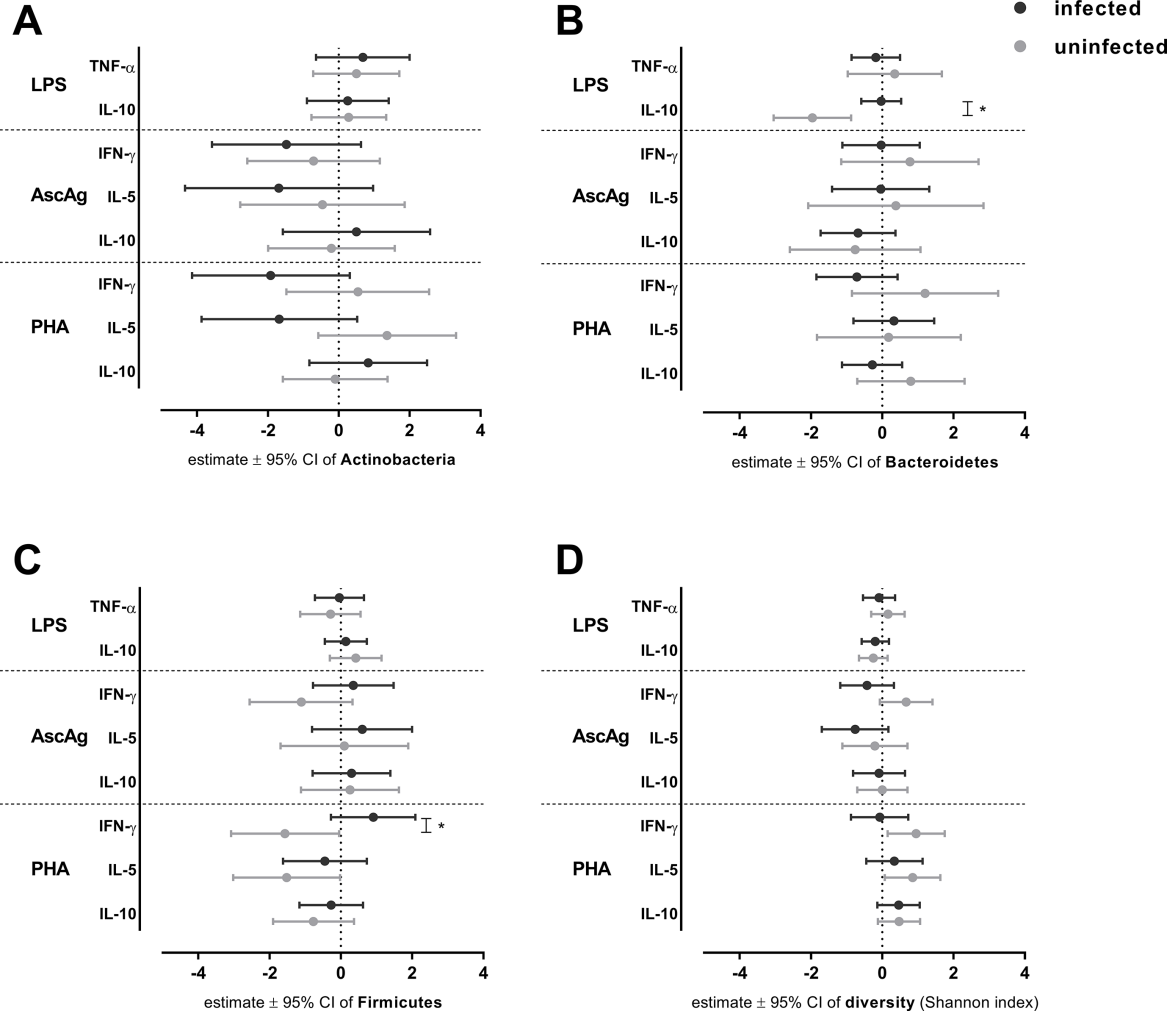
The association between bacterial proportion and diversity on cytokines responses in helminth-negative and -positive subjects. The effect of bacterial proportions on cytokine responses was analyzed for helminth-negative [helminth(−)] and helminth-positive [helminth(+)] groups by a linear mixed model. Estimated effects ± 95% CI are shown for the effect of *Actinobacteria* **(A)** *, Bacteroidetes* **(B)**, *Firmicutes* **(C)** and diversity **(D)** on cytokine responses. For assessing statistical significance modified Bonferroni correction was applied; *p-value ≤ 0.025 (for LPS and PHA responses).

To elucidate the possible role of helminth infections in the interplay of bacteria and immune responses, we conducted analyses in helminth-positive and -negative groups. For this purpose, we used observations at both pre-treatment and post-treatment. Regardless of the randomization arm, we fitted the linear mixed model on each cytokine responses as outcomes. The predictors were bacterial proportions and its interaction with helminth infection. A similar analysis was performed to estimate the association between bacterial diversity and cytokine responses. **Figure 1** illustrates the associations between bacterial proportions or diversity and cytokine responses when subjects were helminth-positive or -negative.

In the innate immune response to LPS, the *Bacteroidetes* proportion showed a significant negative association with IL-10 levels in helminth-negative subjects {estimated effect [95% confidence interval (CI)]: −1.96 (−3.05, −0.87), *p*-value = 0.001; **Figure 1B**} which shows that a unit increase of *Bacteroidetes* proportion will reduce the concentration of IL-10 to LPS almost twice as much. This association was significantly different from that of helminth-negative subjects (*p*-value for the difference = 0.002, **Figure 1**) in which the association was absent [−0.03 (−0.59, 0.53)]. The bacterial diversity had no significant association with IL-10 response to LPS (**Figure 1**). With regard to the helminth-specific cytokine responses, none of IFN-γ and IL-5 responses to AscAg were significantly associated with bacterial proportions or diversity (**Figure 1**). In the adaptive responses (PHA), none of the cytokine responses were significantly associated with the bacterial proportion in uninfected subjects (**Figure 1**). Although not significant, we noticed lower levels of IFN-γ to PHA with higher *Firmicutes* proportions [−1.57 (−3.08, −0.05), *p*-value = 0.045; **Figure 1**]. This association between *Firmicutes* proportion with IFN-γ to PHA in uninfected subjects was however significantly different from that in subjects who were infected (*p*-value for the difference = 0.009, **Figure 1**). At the same time, there was a significantly increasing concentration of IFN-γ to PHA among those who were uninfected when bacterial diversity was higher [0.95 (0.15, 1.75), *p*-value 0.022; **Figure 1**], although this association was not significantly different from the helminth-positive group [−0.07 (−0.88, 0.73), *p*-value for the difference = 0.056; **Figure 1**]. A similar negative association of *Firmicutes* was observed in IL-5 responses to PHA in uninfected subjects [−1.52 (−3.02, −0.02), *p*-value = 0.05; **Figure 1**]. Conversely increasing bacterial diversity led to slightly higher levels of IL-5 to PHA in the uninfected subjects [0.85 (0.07, 1.63), *p*-value = 0.034; **Figure 1**]. Both observations were not significantly different from the effects in those who were helminth-positive.

### The Effect of Albendazole on the Relationship Between Bacterial Proportion and Diversity and *In Vitro* Cytokine Responses

We further investigated whether deworming affects the relationship between bacterial proportions or diversity and cytokine responses. For this purpose, we fitted the linear mixed model on all subjects (n = 66) to characterize the association between bacterial proportions and cytokine responses at two time points and in the two randomization arms. These analyses were irrespective of the infection status. A similar model was applied for the diversity index.

**Figure 2:**
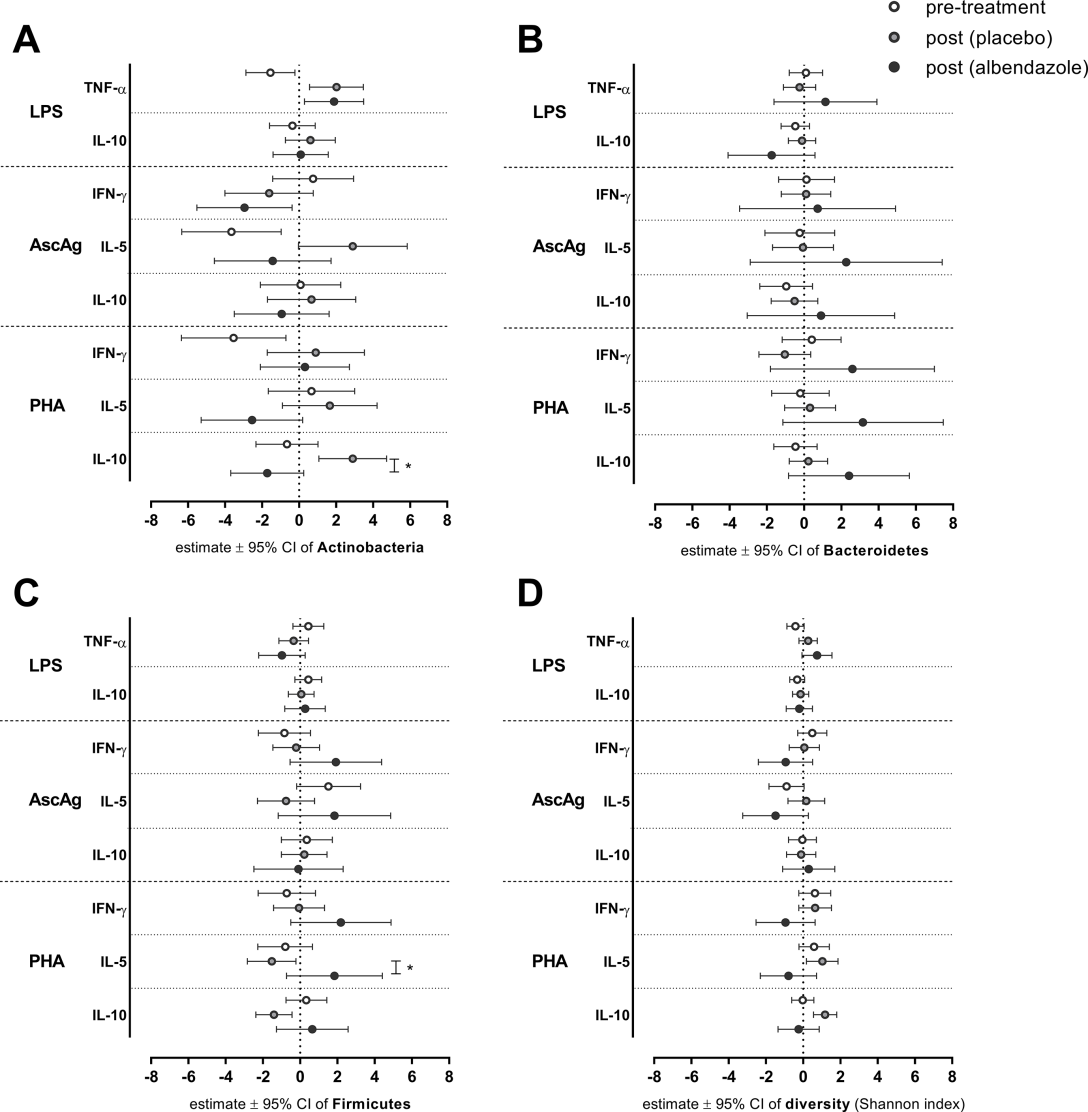
The association between bacterial proportion and diversity on cytokines responses at pre-treatment and post-treatment in two randomization arms. After deworming, comparisons were made for all subjects pre-treatment versus post-treatment (placebo group) and post-treatment placebo versus albendazole groups. Estimated effects of a linear mixed model ± 95% CI are depicted for the effect of Actinobacteria **(A)** *Bacteroidetes* **(B)** *Firmicutes* **(C)** and diversity (Shannon index) **(D)**. *p-value ≤ 0.025 (for LPS and PHA responses) for statistical significance between placebo and albendazole groups.

**Figure 2** displays the associations between the proportions of three major bacterial phyla and diversity with cytokine responses, before and after anthelminthic treatment. With regard to the relationship between *Bacteroidetes* and IL-10 response to LPS, no significant differences were observed between pre- versus post-treatment or between treatment groups (**Figure 2**). While the estimated association between *Bacteroidetes* proportion and IL-10 to LPS at pre-treatment [estimate (95% CI): −0.47 (−1.23, 0.29)] and post-treatment in placebo group [−0.11 (−0.84, 0.62)] were close to zero, the association at post-treatment in albendazole group was clearly lower [−1.74 (−4.08, 0.59); *p*-value for the difference between placebo and albendazole was 0.193, **Figure 2B**]. The association between IFN-γ in response to PHA and bacterial diversity was also not significant at post-treatment either in the placebo or in the albendazole group (**Figure 2D**).

The association between higher *Actinobacteria* proportion with decreasing response of TNF-α to LPS was borderline significant at pre-treatment [estimate (95% CI): −1.55 (−2.87, −0.22), *p*-value = 0.024; **Figure 2A**]. This association was significantly different to the effect of *Actinobacteria* at post-treatment when subject received placebo [2.02 (0.57, 3.47); *p*-value < 0.001; **Figure 2A**], however no difference was observed when comparing placebo and albendazole group [1.89 (0.29, 3.49), *p*-value for the difference = 0.907; **Figure 2A**]. A similar result was obtained from the association between *Actinobacteria* with IL-5 responses to AscAg. At pre-treatment, the increasing *Actinobacteria* proportions were significantly associated with less IL-5 production in response to AscAg [−3.65: (−6.34, −0.97), *p*-value = 0.009; **Figure 2A**]. This association was significantly different to the effect of *Actinobacteria* at post-treatment in placebo group [2.90 (−0.03, 5.84), *p*-value = 0.002]. Although the estimated association in albendazole group was lower [−1.42 (−4.58, 1.73), this was not significantly different between the treatment groups (*p*-value = 0.052)]. The effect of Actinobacteria on IL-10 responses to PHA changed after treatment [−0.65 (−2.33–1.02) vs 2.90 (1.07–4.73) for pre-treatment vs post-treatment placebo group, *p*-value 0.005] and was different between placebo and albendazole groups as well [albendazole group −1.72 (−3.69–0.25), *p*-value for placebo vs albendazole 0.001; **Figure 2A**].

Moreover, while the association between *Firmicutes* and IL-5 response to PHA at pre-treatment was not significantly different compared to the association at post-treatment in placebo group, there was a significant difference of this association between albendazole and placebo group at post-treatment [estimate (95% CI) for placebo −1.52 (−2.83, −0.22) versus albendazole 1.84 (−0.73, 4.41), *p*-value = 0.024; **Figure 2C**].

## Discussion

This study aimed to analyze the effect of helminth infections on the relationship between gut microbiota and the immune system. We found a negative association between proportions of *Bacteroidetes* and IL-10 response to LPS in helminth-negative subjects and the presence of helminths was shown to dampen this effect. Anthelminthic treatment partly recovered this effect, although not statistically significant. To our knowledge, this is the first time that the association between the gut microbiome, presence of parasitic helminths and whole blood cytokine responses was analyzed in a longitudinal study using a randomized placebo-controlled anthelminthic trial.

IL-10 was already marked as a key anti-inflammatory cytokine involved in the induction of immune suppression by helminths (Yazdanbakhsh et al., 2002). Our observation that helminths counteract the suppressed IL-10 response to LPS in subjects with higher *Bacteroidetes* proportions supports the so called “old friends hypothesis” (Rook, 2009), stating that certain infectious agents such as helminths may have protective effects against immune dysfunction and inflammatory diseases, possibly through IL-10. This is strengthened by our observed gradient of the relative abundance of *Bacteroidetes* from rural to urban areas, where immune-related diseases are more prevalent (Bach, 2002). In contrast, a recent meta-analysis indicated that inflammatory bowel disease (IBD) patients displayed lower proportions of *Bacteroidetes* (Zhou and Zhi, 2016), however, this was only found when measuring by real-time quantitative PCR (not by conventional culture) and mainly in Asian studies. Furthermore, a member of the *Bacteroidetes* family, gut inhabitant *Bacteroides fragilis*, was shown to protect mice from experimental colitis, mediated by polysaccharide A (PSA) possibly through IL-10 induction (Mazmanian et al., 2008). However, although *B. fragilis* is the most well-known pathogen of the *Bacteroidetes*, it is the least common species in the *Bacteroidetes* phylum in the human gut (Wexler, 2007). It could therefore be that other factors or species play a dominant role in the general effect of *Bacteroidetes* on IL-10 responses. Further studies are therefore needed to assess the translation of our findings to a clinical setting, for example prevalence or activity of IBD or other auto-immune diseases. Moreover, since we have measured systemic whole blood cytokine responses, we are not sure whether this is representative for the gut responses.

A trend of negative association between *Firmicutes* and concentration of IFN-γ to PHA was seen in helminth-negative subjects only. In subjects with helminth, this association was positive, although this difference fell short of statistical significance. Parallel to this trend, the bacterial diversity was positively associated with IFN-γ responses to PHA in subjects who did not carry helminths, and in helminth-positive subjects, this association was dampened. Since a similar opposite trend was observed in the relationship between *Firmicutes* compared to bacterial diversity on IL-5 responses to PHA, we may conclude that not the proportion of *Firmicutes*, but the total bacterial diversity drove this association. *Firmicutes* was the most abundant phyla in this population and the increasing proportion of *Firmicutes* will obviously reduce diversity. This indicates that analyzing single bacterial phyla without considering the remaining phyla may lead to biased results as microbiome data is compositional and thus correlated between phyla.

Although deworming removed most helminths, treatment did not significantly alter the effects of bacterial proportions on cytokine responses. Regarding the *Bacteroidetes* effect on LPS to IL-10, we did observe a lower effect in the albendazole group compared to placebo. Although not significant, this might point towards the idea that anthelminthic treatment could restore the possibly detrimental- interaction of bacteria with immune responses. Surprisingly, we found differences in immune modulation by *Actinobacteria* in the pre- versus post-treatment group. Although there was a significant association of time (in subjects receiving a placebo), these associations were not significantly different in the albendazole group. The effect of time could be explained by other factors such as diet and possibly improved hygiene, resulting from increased awareness during the presence of our medical team in the study area. In the analysis of treatment’s effect on the association between bacterial proportion and diversity, there was a significant difference between the association of *Firmicutes* on the IL-5 response to PHA in the albendazole group compared to the placebo group. In subjects receiving albendazole, *Firmicutes* proportions were positively associated with IL-5 levels, while we observed a negative (non-significant) effect in helminth-negative individuals over time. This result seems contradictory, but might be related to the fact that small numbers were analyzed and not everyone in the albendazole group lost their helminth infection. The analysis of subjects who were infected at baseline and cleared their infection would possibly reveal more clearly how the relationship between bacterial communities and immunity are affected by treatment. This analysis lacks statistical power in our study as the sample size was small (n = 12 out of 17 subjects who were successfully dewormed). Future research which involves larger sample sizes needs to be conducted. With larger sample size, the current statistical model can be extended to account for different infection status at different time points as well as different randomization arms. Thereby we would gain more insight in the responses within individuals, and how these are affected by worm infection and deworming. Another relevant thought in this and similar research settings is that although albendazole removes helminths effectively, the immunomodulatory effects of helminths on cytokine responses are long-lasting and cannot be easily corrected by short-term treatment. It was previously reported by Endara et al. that the length of periodic treatment is important for altering immune responses (Endara et al., 2010), i.e. that studies with a longer period of treatment (up to 30 months) are more likely to show effects of deworming.

As significant associations between bacterial communities and cytokine responses were only observed when subjects were helminth-negative, clearly other factors than helminth and treatment are also involved in the alteration of the microbiome community and their interaction with the immune system. For example, our study data lack information on diet. Dietary intake was clearly shown to affect bacterial communities in the gastrointestinal tract (Wu et al., 2011). This might also be related to changes in social economic status leading towards a more high-fat diet when moving from rural to urbanized areas. Recent articles reported inconsistencies with regard to the direction of *Bacteroidetes* to *Firmicutes* ratio in rural to urban comparisons of microbiome profiles from different geographical areas. Studies comparing children from Bangladesh to USA children showed the direction of increasing Bacteroidetes: *Firmicutes* in the USA, as observed in our data (Lin et al., 2013), while studies in elderly Korean and children in Burkina Faso showed opposing results, i.e. decreasing *Bacteroidetes*: *Firmicutes* ratios from rural to urban (Park et al., 2015; Filippo et al., 2017). This could be caused by different genera under *Bacteroidetes* or *Firmicutes* phyla which might be affected by certain type of diet. Therefore, it will be beneficial for future studies to also include dietary factors from the study participants.

A further limitation is related to the statistical tools available in analyzing this relationship. Here, we characterized the association of three single bacterial proportions on cytokine response in the helminth-positive and -negative group. Using this approach, we first ignore the effect of compositional structure in the microbiome data, namely when computing the *p*-value we assumed that these bacterial categories are independent while they are correlated. Secondly, the current statistical model ignores the fact that microbiome is a variable measured with errors at a different scale than the cytokine responses (Teixeira-Pinto et al., 2009). In addition, we might as well ignore the possible unobserved confounders. It is therefore important for future studies in this field to develop a statistical method to characterize the effects of helminth infection on both outcomes simultaneously by accounting these unobserved errors with a joint model.

To conclude, our findings support the hypothesis for the role of helminths in modulating the immune response, which might be related to bacterial proportion and diversity. Deworming did not show a particular effect on the observed associations. It is therefore important to repeat such studies with a larger sample size as well as using more advanced statistical models to further analyze this relationship by considering the complex structure of microbiome data and other possible confounders.

## Data Availability Statement

The microbiome datasets generated during the current study are available at the NCBI’s Sequence Read Archive (SRA) accession numbers: SAMN07688522 to SAMN07688545. The ∼ 4 million 16S assembled sequences from Indonesia samples are also available for download from Nematode.net (Nematode.net/Microbiome.html). The datasets supporting the conclusions of this article are available in the following Github address: https://github.com/IvonneMartin/Helminths_GutMicrobes_Cytokine.

## Ethics Statement

This study was nested within the ImmunoSPIN study, a double-blind placebo-controlled trial conducted in Flores Island, Indonesia (Wiria et al., 2010). The ImmunoSPIN study has been approved by the Ethical Committee of Faculty of Medicine, Universitas Indonesia, ref:194/PT02.FK/Etik/2006 and has been filed by ethics committee of the Leiden University Medical Center. The clinical trial was registered with number: ISRCTN83830814. The subjects gave their informed consent either by written signature or thumb print. Parental consent was obtained for children below 15 years old.

## Author Contributions

IM, JH-D, and LW performed the analyses and wrote the manuscript. MK, AW, FH, YD, ES, and LW performed research on cytokine data. BR performed the microbiome data processing. TS, MM, and MY designed the study. All authors read, edited and approved the final manuscript.

## Funding

This study was funded by The Royal Netherlands Academy of Arts and Science (KNAW), Contract: 57-SPIN3-JRP. Sequencing data generation was supported by National Institutes of Health (NIH), grant number U54HG003079 and AI081803. The doctoral research of IM was supported by joint scholarships from the Directorate General of Resources for Science Technology and Higher Education (DGRSTHE) of Indonesia – Leiden University. The findings and conclusions contained within are those of the authors. The funders had no role in data collection, analysis, and interpretation of the data, had no role in the writing of the manuscript and in the decision to publish.

## Conflict of Interest

The authors declare that the research was conducted in the absence of any commercial or financial relationships that could be construed as a potential conflict of interest.
