# Supplementary material for: The Effect of Gut Microbiome Composition on Human Immune Responses: An Exploration of Interference by Helminth Infections
Source: Front Genet. 2019 Nov 6;10:1028. doi: 10.3389/fgene.2019.01028 (PMC6856646; doi:10.3389/fgene.2019.01028)
Supplement: Supplementary file 1 [file Table_1.docx]

**Table S1. The comparison between the 66 selected subject with cytokine data vs 150 subjects with only microbiome data**

| **Characteristics** | **Total Sample (N=150)** | **Selected Samples (N=66)** |
| --- | --- | --- |
|  |  |  |
|  |  |  |
| Age (in years), mean (SD) | 27.6 (16.7) | 27 (15.7) |
| Sex, female, n(%) | 84 (56) | 34 (51.5) |
| **Proportion (in %) of the 6 most abundant bacteria** |  |  |
| **phyla, mean(SD)** |  |  |
| *Actinobacteria* | 11.7 (8.4) | 9.4 (7.0) |
| *Bacteroidetes* | 6.9 (11.2) | 7.5 (11.4) |
| *Firmicutes* | 68.6 (13.6) | 71.9 (11.8) |
| *Proteobacteria* | 9.4 (8.1) | 8.5 (6.6) |
| Unclassified | 2.3 (2.8) | 2.1 (2.5) |
| Pooled | 1.1 (2.7) | 0.6 (0.9) |
