# Supplementary material for: The Effect of Gut Microbiome Composition on Human Immune Responses: An Exploration of Interference by Helminth Infections
Source: Front Genet. 2019 Nov 6;10:1028. doi: 10.3389/fgene.2019.01028 (PMC6856646; doi:10.3389/fgene.2019.01028)
Supplement: Supplementary file 2 [file Table_2.docx]

**Table S2** **The characteristics of the participants at 21 months after treatment.**

| Characteristics | albendazole | | placebo | |
| --- | --- | --- | --- | --- |
|  | N | Result | N | Result |
| **Parasite infection(%)** |  |  |  |  |
| *A. lumbricoides* | 26 | 3 (11.5) | 40 | 17 (42.5) |
| Hookworm | 26 | 0 (0) | 40 | 11 (27.5) |
| *N. americanus* | 26 | 0 (0) | 40 | 11 (27.5) |
| *A. duodenale* | 26 | 0 (0) | 40 | 2 (5.0) |
| *T. trichiura* | 26 | 4 (15.4) | 40 | 13 (32.5) |
| Any helminths | 26 | 5 (19.2) | 40 | 26 (65.0) |
| **Proportion (in %) of the 6 most abundant bacteria phyla, mean(SD)** | 26 |  | 40 |  |
| *Actinobacteria* |  | 14.1 (8.9) |  | 9.2 (7.4) |
| *Bacteroidetes* |  | 3.6 (5.8) |  | 7.7 (14.1) |
| *Firmicutes* |  | 60.1 (13.7) |  | 59.2 (16.7) |
| *Proteobacteria* |  | 9.0 (6.5) |  | 8.7 (6.8) |
| Unclassified |  | 1.8 (1.1) |  | 2.6 (2.8) |
| Pooled |  | 11.5 (6.7) |  | 12.5 (7.4) |
| **Diversity Index, median(IQR)** | 26 |  | 40 |  |
| Shannon index |  | 0.90 (0.85, 1.08) |  | 0.97 (0.79, 1.05) |
| Bray-Curtis |  | 0.19 (0.14, 0.26) |  | 0.24 (0.17, 0.36) |
